# Effects of music interventions on disorders of consciousness: a systematic review and meta-analysis

**DOI:** 10.3389/fnins.2026.1843996

**Published:** 2026-07-24

**Authors:** Aoyi Li, Huimin He, Yingcong Liu, Qiyu Jiang, Yupeng He

**Affiliations:** Wuhan Conservatory of Music, Wuhan, Hubei, China

**Keywords:** coma, disorders of consciousness, meta-analysis, music intervention, systematic review

## Abstract

**Objective:**

The study aimed to systematically evaluate the effects of music intervention on outcomes related to the recovery of consciousness in patients with disorders of consciousness (DOC).

**Methods:**

Databases including PubMed, Web of Science, Embase, the Cochrane Library, China National Knowledge Infrastructure (CNKI), and ProQuest were systematically searched from January 1970 to December 2025 to identify relevant randomized controlled trials (RCTs). The literature was screened based on pre-established inclusion and exclusion criteria, and the review was reported in accordance with the Preferred Reporting Items for Systematic Reviews and Meta-Analyses (PRISMA) guidelines. The methodological quality of the included studies was assessed using the Cochrane Risk of Bias tool (RoB 1).

**Results:**

A total of 12 randomized controlled trials (RCTs) involving 926 patients with disorders of consciousness (DOC) were included. As the types of outcome indicators reported in the included studies varied, the number of studies included for each outcome also differed. Specifically, five studies were included in the analyses of Glasgow Coma Scale (GCS) scores and the GCS-based awakening rate, whereas two studies were included in the analyses of GCS scores of > 8, the Coma Recovery Scale-Revised (CRS-R), and the National Institutes of Health Stroke Scale (NIHSS). A meta-analysis showed that GCS scores were significantly higher in the music intervention group than in the control group (mean difference (MD) = 2.32). The analyses of GCS scores of > 8 showed that a higher proportion of patients in the music intervention group achieved a GCS score of > 8 (odds ratio (OR) = 5.98). The awakening rate also indicated that the music intervention group was significantly superior to the control group (OR = 3.76). For secondary outcomes, the music intervention group showed higher CRS-R scores (MD = 2.86) and lower NIHSS scores (MD = −1.24).

**Conclusion:**

Music intervention may help improve consciousness levels and awakening-related outcomes in patients with disorders of consciousness and may serve as a safe and non-invasive adjunctive measure in rehabilitation nursing care. However, more high-quality randomized controlled trials are needed to further verify its effects and mechanisms.

**Systematic review registration:**

https://www.crd.york.ac.uk/prospero/display_record.php?ID=CRD420261354569, Identifier: CRD420261354569.

## 1 Introduction

Disorders of consciousness (DOC) refer to a spectrum of clinical conditions that occur after severe brain injury and are characterized by impaired wakefulness and/or consciousness (Giacino et al., 2018). These disorders are commonly observed in patients with brain damage resulting from traumatic brain injury, stroke, hypoxic encephalopathy, and other conditions (Kondziella et al., 2020). These conditions generally include coma, vegetative state/unresponsive wakefulness syndrome (VS/UWS), and minimally conscious state (MCS) (Giacino et al., 2018; Kondziella et al., 2020). Moreover, these conditions not only restrict the patient’s self-awareness and responsiveness but also impose immense pressure on families and society, significantly affecting the patient’s daily life and rehabilitation progress.

Currently, conventional interventions for disorders of consciousness (DOC) primarily include pharmacotherapy, surgery, neuromodulation techniques, and rehabilitation training. While certain medications and interventions can alleviate symptoms to some extent, the efficacy of current treatments often remains unpredictable due to individual variability. Pharmacotherapy, particularly when administered long term, may lead to drug resistance, dependence, and other adverse effects (Dutta et al., 2024), necessitating particular caution in older patients. Although methods such as physical rehabilitation and hyperbaric oxygen therapy (HBOT) facilitate recovery in some patients, their therapeutic efficacy is also limited. Consequently, exploring a safe, effective, and sustainable therapeutic modality has become a prominent research focus in the medical field.

Notably, “music intervention” is an umbrella term that utilizes music as a therapeutic medium, generally encompassing various implementation approaches, such as music therapy, music medicine, and “musical stimulation,” which are commonly found in the literature (Bradt et al., 2016; Li, 2016). Among these approaches, music therapy emphasizes interventions implemented by professionally trained and qualified music therapists within a therapeutic relationship to achieve individualized treatment goals (Li, 2016; American Music Therapy Association, 2005); in contrast, music medicine primarily refers to the listening of pre-recorded music implemented by non-music therapy professionals, such as physicians, nurses, or researchers, without the involvement of a music therapist (Bradt et al., 2016; Ginsberg et al., 2022). Conversely, musical stimulation is more accurately described as a specific operational method that typically manifested as passive listening with music playback as the core stimulatory approach. In the absence of a music therapist, its implementation at the operational level is largely similar to that of music medicine (Li, 2016). Based on this, the present study used “music intervention” as a collective term during data encoding and further categorized the included studies into music therapy, music medicine, or musical stimulation based on the implementer and the method of implementation to enhance the clarity of comparisons across different studies.

Previous studies have suggested that music intervention may promote the emergence of behavioral responses in patients with disorders of consciousness through pathways involving emotional responses, cognitive responses, and autonomic nervous regulation (Riganello et al., 2015). At the neural mechanism level, musical stimulation can activate the frontoparietal network, the superior temporal gyrus, and the limbic system (Okumura et al., 2014) (e.g., the amygdala and hippocampus) and may enhance functional connectivity within the default mode network (Heine et al., 2015). Furthermore, music intervention can evoke immediate behavioral responses (Binzer et al., 2016) (such as increased eye-opening frequency) by regulating autonomic nervous activity, thereby synergistically promoting the recovery of consciousness. Overall, these studies provide a theoretical basis for using music intervention to promote awakening and rehabilitation in patients with disorders of consciousness; however, its clinical effects remains to be further evaluated based on evidence from randomized controlled trials (RCTs).

However, relevant studies exhibit significant heterogeneity in intervention methods and outcome measures, and comprehensive evidence for patients with DOC remains insufficient, necessitating further research. A previous study conducted a systematic review in this field but did not perform a meta-analysis (Grimm and Kreutz, 2018); another meta-analysis attempted to quantitatively synthesize existing evidence; however, its included studies were not strictly limited to randomized controlled trials (RCTs) (Li et al., 2020). Due to the fundamental differences in bias control and evidence strength between RCTs and other study designs, merging different designs—while expanding the sample source—increases the complexity of result interpretation and weakens the robustness of the conclusions. Furthermore, previous English-language reviews have provided relatively limited coverage of the Chinese literature (Li et al., 2020; Rollnik, 2014), whereas several RCTs on music intervention for DOC patients have recently been reported in both Chinese and English. Consequently, the present study exclusively included RCTs of music intervention for DOC patients, systematically searching international databases while supplementing with Chinese databases such as China National Knowledge Infrastructure (CNKI). A systematic review and meta-analysis were performed on outcomes, including the Glasgow Coma Scale (GCS), GCS > 8, awakening rate, Coma Recovery Scale-Revised (CRS-R), and National Institutes of Health Stroke Scale (NIHSS). Considering that the included studies comprised both two-arm RCTs and three-arm trials with different control structures, this study aimed to provide a more objective evaluation of the clinical value of music intervention for DOC patients within a more consistent evidence framework. It also seeks to provide a basis for future research to further clarify the differences in efficacy across various musical materials, implementation methods, and control conditions.

## 2 Methods

### 2.1 Registration and protocol

This systematic review was prospectively registered in PROSPERO (CRD420261354569).

### 2.2 Search strategy

This study utilized a systematic review methodology by searching six major databases—Web of Science, PubMed, Embase, the Cochrane Library, ProQuest, and China National Knowledge Infrastructure (CNKI)—for studies on music intervention for disorders of consciousness (DOC) published between January 1970 and December 2025. The search was limited to the English and Chinese languages, with Boolean logic operations executed through advanced search functions. The search string for the Web of Science was as follows: TS = (“Consciousness Disorders” OR “Consciousness Disorder” OR “Disorders of Consciousness” OR “Disorder of Consciousness” OR “Consciousness, Level Depressed” OR “Depressed Level of Consciousness” OR “Semiconsciousness” OR “Consciousness, Level Altered” OR “Altered Level of Consciousness” OR “Coma” OR “Comas” OR “Comatose” OR “PseuDOComa” OR “PseuDOComas” OR “Persistent Vegetative State” OR “PVS” OR “PVSs” OR “Persistent Vegetative States” OR “Vegetative State, Persistent” OR “Vegetative States, Persistent” OR “Permanent Vegetative State” OR “Permanent Vegetative States” OR “Vegetative State, Permanent” OR “Vegetative States, Permanent” OR “Vegetative State” OR “Vegetative States” OR “Transient Vegetative State” OR “Transient Vegetative States” OR “Minimally Conscious State” OR “Minimally Conscious States” OR “MCS” OR “Unresponsive Wakefulness Syndrome” OR “UWS”) AND TS = (“Music” OR “Music Therapy” OR “Therapy, Music” OR “Rap Music” OR “Music, Rap” OR “Hip Hop Music” OR “Hop Music, Hip” OR “Music, Hip Hop” OR “Jazz Music” OR “Music, Jazz” OR “Classical Music” OR “Music, Classical” OR “Songs” OR “Song” OR “Vocal Melody” OR “Melodies, Vocal” OR “Melody, Vocal” OR “Vocal Melodies” OR “Rock and Roll Music”). Similar logical frameworks were applied to the other databases. To ensure methodological rigor, the search process strictly followed the Preferred Reporting Items for Systematic Reviews and Meta-Analyses (PRISMA) 2020 Statement guidelines. A multilingual search strategy was employed to improve the comprehensiveness of the literature search to the greatest extent possible.

### 2.3 Eligibility criteria

The inclusion criteria were as follows: (1) Study population: Adult patients (≥18 years) with acquired brain injury-related disorders of consciousness (DOC), including coma, vegetative state/unresponsive wakefulness syndrome (VS/UWS), and minimally conscious state (MCS). Original studies that did not clearly distinguish specific DOC subtypes but described participants as having disorders of consciousness or impaired levels of consciousness were also included if they reported extractable consciousness-related outcomes in patients with acquired brain injury. Etiologies were not restricted, including traumatic brain injury, cerebral hemorrhage, cerebral infarction, or other acquired brain injuries. Studies focusing primarily on neurodegenerative diseases, such as dementia, were excluded. (2) Intervention: The experimental group received interventions centered on music. To enhance encoding consistency, “music intervention” was used as an umbrella term and operationally categorized into music therapy, music medicine, or musical stimulation based on the implementer and the method of implementation. (3) Comparison: The control group could receive routine treatment, routine care, standard care, no auditory stimulation/placebo auditory control, or other non-target music/non-target auditory stimulation. Multi-arm trials were included if they comprised at least one music intervention group and one comparable control group, as defined in this study. (4) Outcome measures: At least one clinical outcome related to arousal levels, awakening outcomes, or neurological status was reported, such as the Glasgow Coma Scale (GCS), GCS score > 8, awakening rate, Coma Recovery Scale-Revised (CRS-R), or National Institutes of Health Stroke Scale (NIHSS). Among these measures, the NIHSS was included as a supplementary outcome to reflect the degree of neurological deficit. (5) Study design: Randomized controlled trials (RCTs), including two-arm parallel RCTs and multi-arm RCTs, were eligible. For studies with three or more arms, they were retained as a single study entry in the encoding table. In the meta-analysis, when multiple experimental arms met the definition of music intervention and required comparison with the same control group, these experimental arms were preferably merged into a single comparison; if separate arm comparisons were maintained, the shared control group was split according to a preset approach to avoid double-counting. (6) Literature type and language: Eligible literature included peer-reviewed, full-text publications in Chinese or English.

The exclusion criteria were as follows: (1) duplicate publications; (2) reviews, protocols, case reports, theoretical articles, conference abstracts, dissertations, and other non-peer-reviewed literature; (3) non-randomized controlled trials (non-RCTs), such as single-group pre–post studies, quasi-experimental studies, cohort studies, or case–control studies; (4) studies in which music intervention was so closely combined with other interventions that the independent effect of music could not be isolated; (5) studies that did not report extractable outcome data, or those with insufficient full-text information that remained unresolvable after verification; and (6) studies in which the participants were not DOC patients or the study population did not match the study target, such as children or patients with neurodegenerative diseases.

Primary outcomes: Primary outcomes included the Glasgow Coma Scale (GCS) (Teasdale and Jennett, 1974), awakening rate, and Coma Recovery Scale-Revised (CRS-R) (Giacino and Kalmar, 2004). Supplementary outcomes: Supplementary outcomes included measures reflecting the degree of neurological impairment, such as the National Institutes of Health Stroke Scale (NIHSS) (Zhang and Liu, 2014).

### 2.4 Data extraction

Zotero software was used to identify and manually remove duplicate records. Based on the inclusion and exclusion criteria, titles and abstracts were first screened to exclude obviously ineligible studies, followed by a full-text review to further determine eligibility. In total, two reviewers independently extracted encoded information, including author, year of publication, sample size, experimental group interventions, control group interventions, music source, music form, and outcome measures. Any disagreements were resolved through discussion and adjudicated by a third reviewer to ensure the accuracy of study selection and data extraction.

### 2.5 Risk of bias assessment

In total, two reviewers independently assessed the methodological quality of the included studies using the Risk of Bias tool (RoB 1) recommended by the Cochrane Handbook for Systematic Reviews of Interventions. The assessment domains included random sequence generation (selection bias), allocation concealment (selection bias), blinding of participants and personnel (performance bias), blinding of outcome assessment (detection bias), incomplete outcome data (attrition bias), selective reporting (reporting bias), and other bias. Each item was categorized as “low risk,” “high risk,” or “unclear risk.” Any disagreements between the two reviewers were resolved through discussion, with a third reviewer adjudicating if necessary.

### 2.6 Certainty of evidence assessment

The GRADE approach was used to assess the certainty of evidence for each outcome included in the meta-analysis, with the evaluation time point defined as the measurement closest to the end of the intervention (Guyatt et al., 2011). Given that all included studies were randomized controlled trials (RCTs), the initial certainty of evidence for each outcome was rated as “high.” Subsequently, assessments were conducted across the five downgrading domains recommended by GRADE, including risk of bias, inconsistency, indirectness, imprecision, and publication bias (Guyatt et al., 2011; Schünemann et al., 2011). If a serious concern was identified in one domain, the certainty of evidence was downgraded by one level; if a very serious concern was identified, it was downgraded by two levels. Downgrading could be cumulative when concerns were present across multiple domains, and the final certainty of evidence was categorized into four levels: high, moderate, low, and very low. Specifically, risk of bias was judged primarily based on the RoB 1 results; inconsistency was assessed by considering the direction of effects, the magnitude of heterogeneity (*I*^2^), and its interpretability across pooled analyses for each outcome. Indirectness was assessed by examining the consistency of participants, interventions, comparators, and outcome measures with the research question; imprecision was assessed based on sample size, number of events, and the width of the 95% confidence interval (95% CI); and publication bias was prudently judged based on the number of included studies, distribution of sample sizes, direction of results, and proportion of small-sample studies. GRADE assessments were independently performed by two reviewers, and disagreements were resolved through discussion or, when necessary, adjudication by a third reviewer.

### 2.7 Statistical analysis

For continuous outcomes, mean differences (MDs) or standardized mean differences (SMDs) with 95% confidence intervals (95% CIs) were used as effect sizes; for binary outcomes, odds ratios (ORs) with 95% CIs were used. Inter-study heterogeneity was assessed using Cochran’s *Q* test and the *I*^2^ statistic. A fixed-effects model was applied when *I*^2^ was ≤ 50% and *p* was ≥ 0.10; otherwise, a random-effects model was used (*I*^2^ > 50% or *p* < 0.10). Given the variations in outcome definitions and reporting across studies, separate pooled analyses were conducted for the primary outcome indicator, namely the Glasgow Coma Scale (GCS). These analyses included continuous GCS scores, binary awakening rate outcomes based on the GCS, and binary outcomes using a GCS score of > 8 as the threshold. The awakening rate, a binary outcome, was defined as the proportion of patients classified as awake based on clinical judgment or improvement in the GCS that met the prespecified criteria in the original studies. Since definitions of awakening may vary across studies, data were extracted according to the original definition and analyzed separately. For multi-arm RCTs, when multiple experimental arms were included in the same outcome analysis against a single control group, similar music intervention arms were preferably merged; when separate comparisons were required, the sample size of the shared control group was divided to avoid unit-of-analysis errors. Sensitivity analysis was performed for outcomes with a relatively large number of studies using the leave-one-out method; for outcomes with few studies, sensitivity analysis served only as a supplementary assessment and was not presented separately.

## 3 Results

### 3.1 Search results

**Figure 1:**
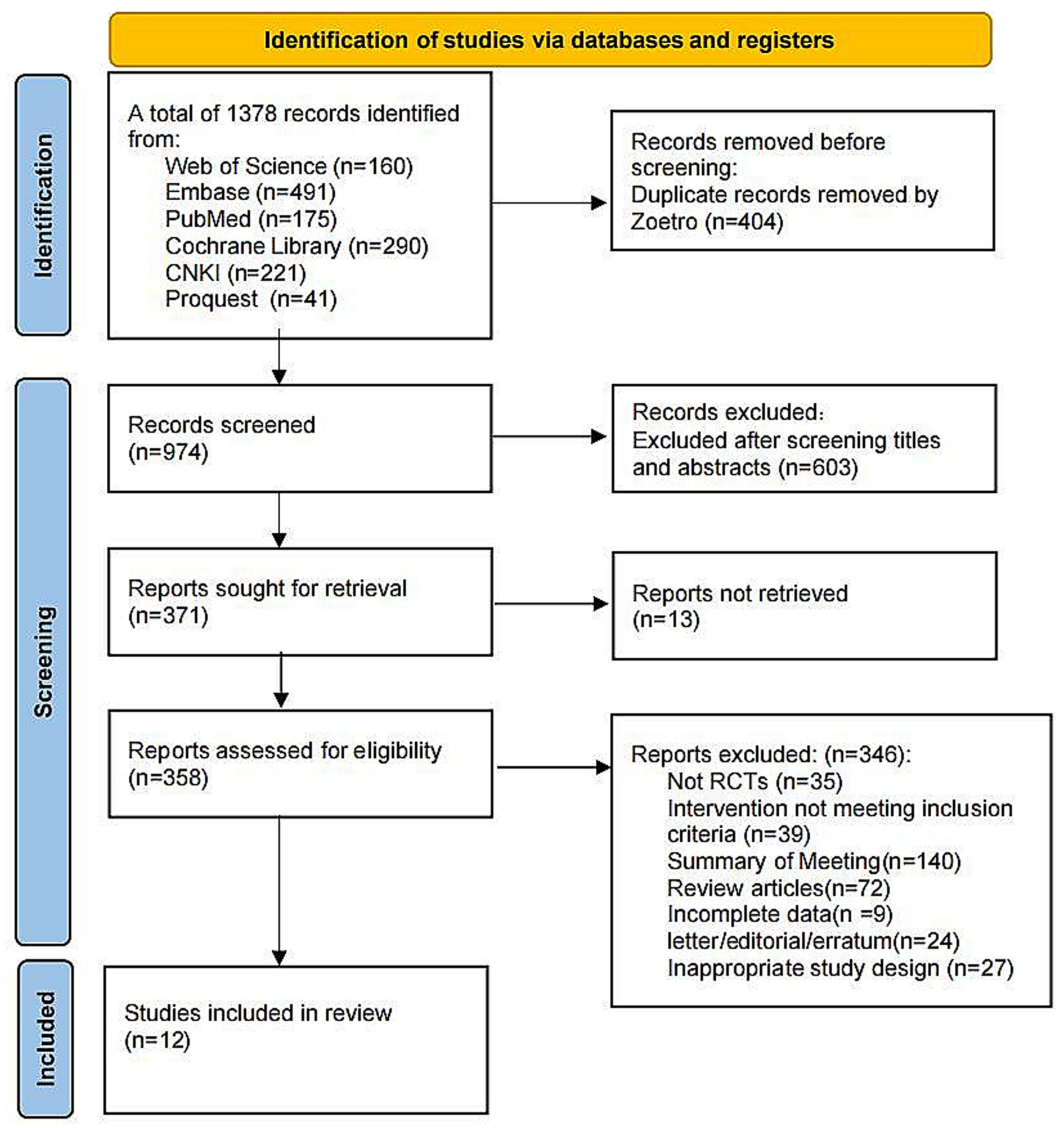
Flowchart of the literature selection process.

After screening, a total of 12 studies in Chinese and English were finally included (Hao et al., 2024; Jia et al., 2013; Yekefallah et al., 2021; Li et al., 2019; Xiao et al., 2023; Dai et al., 2016; Yu et al., 2022; Chen and Wang, 2011; Liang, 2008; Liang, 2005; Xu et al., 2019; Zhu et al., 2019), involving 926 participants, with 447 in the control group and 479 in the experimental group. The literature screening process is illustrated in Figure 1, and the basic characteristics and DOC subtypes of the included studies are summarized in Table 1.

**Table 1:** Characteristics of the included studies.

Author	DOC subtype	Sample (treat/control)	Interventions				Outcome
			Experimental	Control	Source	Form	
Yekefallah (2020)	Impaired LOC-TBI	54 (27/27)	Musical stimulation + routine treatment	Silent headphones + routine treatment	Selected by healthcare staff	Playback	①
Xiao (2023)	MCS	15 (5/5 + 5)	Music therapy + routine treatment	Family voice stimulation + routine treatment; routine treatment	Experimental group: Performed by the therapist; Control 1: Provided by family members; Control 2: None	Experimental group: Live; Control 1: Playback	④
Xijun (2024)	Coma-TBI	70 (35/35)	Music medicine + routine treatment	Routine treatment	Selected by healthcare staff	Playback	①③④
Shuping (2019)	DOC-NOS-CI	102 (51/51)	Music medicine + routine pharmacotherapy	Routine pharmacotherapy	Selected by healthcare staff	Playback	⑤
Hongying (2008)	Coma-ICH	60 (30/30)	Music medicine + other interventions + routine treatment	Routine treatment	Provided by family members	Playback	①
Ying (2005)	Coma-TBI	120 (60/60)	Music medicine + routine treatment	Routine treatment	Provided by family members	Playback	①③
Feifeng (2008)	Coma-TBI	130 (65/65)	Music medicine + routine treatment	Routine treatment	Provided by family members	Playback	①③
Chunmei (2019)	DOC-NOS-ICH	86 (43/43)	Music medicine + routine nursing care	Routine nursing care	Provided by family members	Playback	⑤
Aiqin (2019)	DOC-NOS-TBI	60 (30/30)	Music medicine + routine treatment	Routine treatment	Selected by healthcare staff	Playback	①③④
Jie (2013)	DOC-NOS-ICH	40 (20/20)	Music medicine + routine treatment	Routine treatment	Selected by healthcare staff	Playback	①
Yujian (2022)	Impaired LOC-TBI	80 (40/40)	Music medicine + routine nursing care	Routine nursing care	Provided by family members	Playback	②
Minchao (2016)	DOC-NOS-TBI	109 (36 + 37/36)	Experimental arm 1: Music medicine + routine treatment; Experimental arm 2: Music medicine + routine treatment	Control arm: Routine treatment	Experimental arm 1: Provided by family members; Experimental arm 2: Selected by healthcare staff	Playback	②

(A) The DOC subtype was coded according to the diagnostic description reported in each original study. MCS, minimally conscious state; LOC, level of consciousness; DOC-NOS, disorders of consciousness-not otherwise specified; this label indicates that the original study reported disorders of consciousness or consciousness disturbance but did not clearly distinguish coma, VS/UWS, and MCS; TBI, traumatic brain injury; CI, cerebral infarction; ICH, intracerebral hemorrhage. (B) “Sample” indicates the total number of participants included in the study, with the following row representing the number of participants in the experimental and control groups, respectively. For three-arm studies, the sample size is recorded in the format of (experimental arm 1 + experimental arm 2)/control arm or experimental arm/(comparison arm 1 + comparison arm 2). (C) The “Experimental” column records the interventions received by the experimental group. To enhance coding consistency, “music intervention” was used as an umbrella term and further subdivided into musical stimulation, music therapy, and music medicine based on the implementer and the method of implementation. If the original study explicitly labeled the intervention type, the original terminology was retained; otherwise, operational classification was performed based on the implementation subject, intervention process, and presentation mode. (D) “Form” refers to the implementation format of the music intervention, namely the method of music playback or presentation; for three-arm studies, these are labeled according to the actual source for each arm. (E) ① Glasgow Coma Scale (GCS); ② GCS > 8; ③ Awakening rate; ④ Coma Recovery Scale-Revised (CRS-R); and ⑤ National Institutes of Health Stroke Scale (NIHSS). (F) According to the diagnostic descriptions reported in the original studies, four studies explicitly investigated comatose patients, one study investigated patients with minimally conscious state (MCS), and no study specifically focused on unresponsive wakefulness syndrome (UWS). The remaining seven studies reported impaired levels of consciousness, consciousness disturbance, or broadly defined disorders of consciousness without clearly distinguishing coma, VS/UWS, and MCS.

### 3.2 Risk of bias assessment

**Figure 2:**
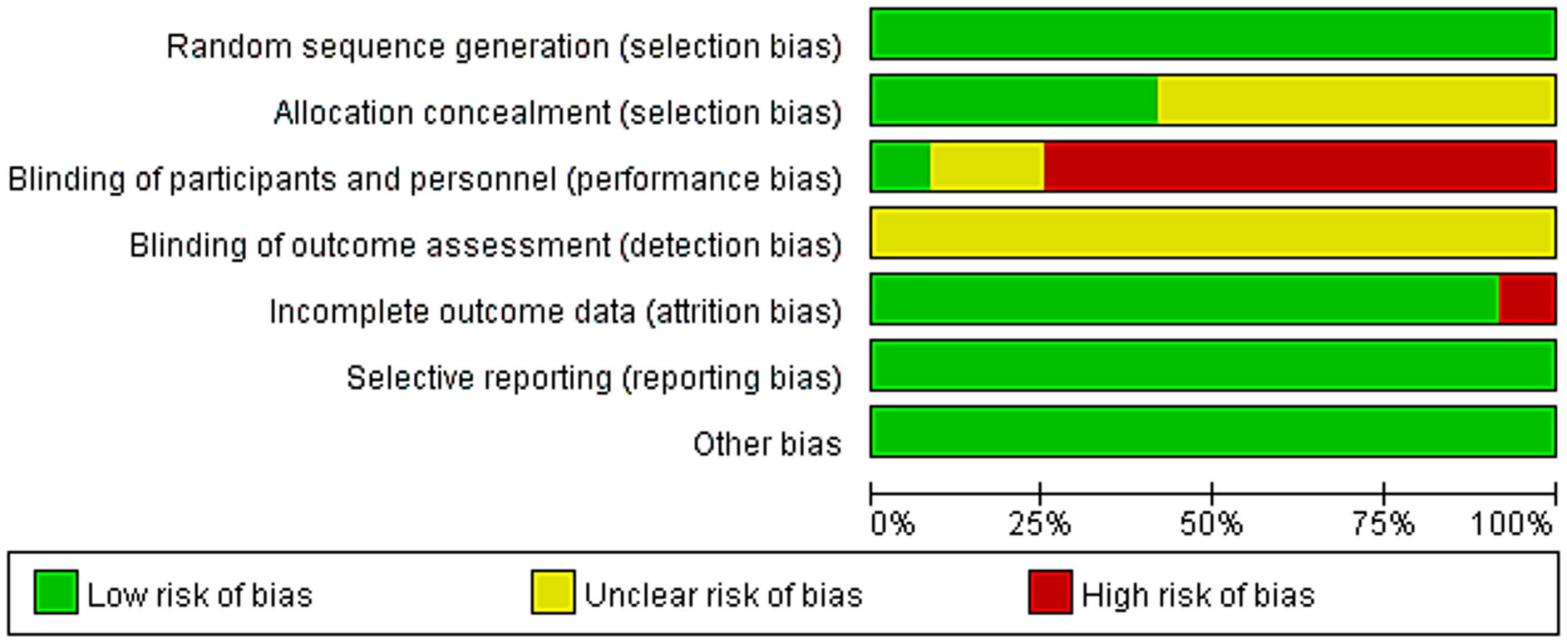
Risk-of-bias assessment of the included studies.

**Figure 3:**
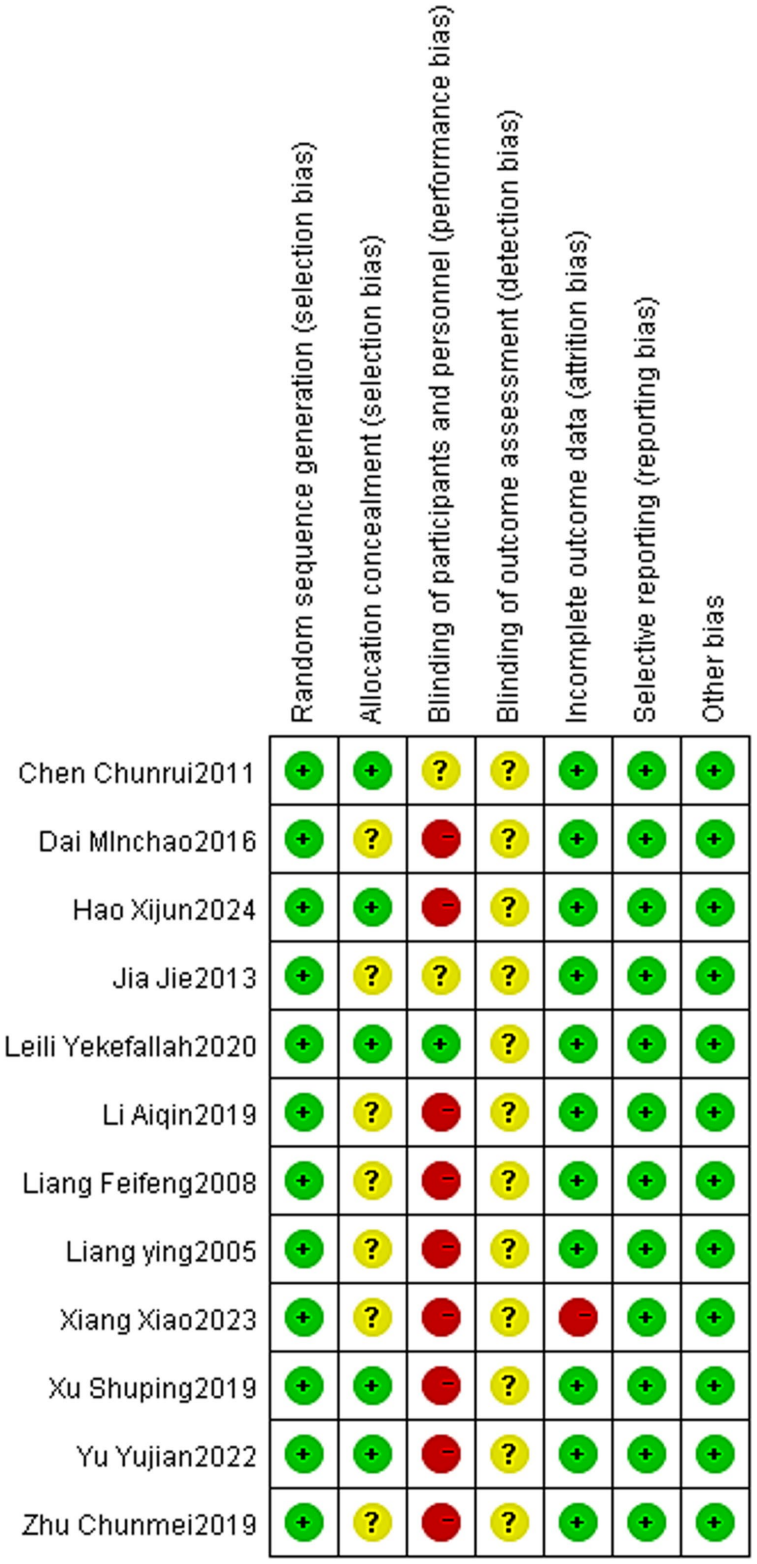
Risk-of-bias graph of the included studies.

The methodological quality of the included studies was assessed using the Cochrane Risk of Bias tool (RoB 1). Among the 12 included studies (see Figures 2, 3), five reported appropriate randomization methods, including the use of the random number table method. Only one study reported blinding of outcome assessors, while the remaining studies did not mention blinding procedures. One study reported loss to follow-up, whereas outcome data were complete in the remaining studies. No other obvious sources of bias were identified.

### 3.3 Meta-analysis results

#### 3.3.1 Effects of music intervention on consciousness-related outcomes assessed by the GCS and awakening rate

Due to variations in how consciousness improvement outcomes were reported across the included studies, some studies used continuous Glasgow Coma Scale (GCS) scores, while others used a GCS score of > 8 as a threshold or reported awakening rates. To minimize clinical heterogeneity arising from different outcome definitions, stratified pooled analyses were conducted for each outcome type.

The analysis of continuous GCS scores included five studies, with 115 patients in the experimental group and 115 in the control group. Heterogeneity testing indicated low inter-study heterogeneity (*χ*^2^ = 5.07, df = 4, *p* = 0.28; *I*^2^ = 21%); therefore, a fixed-effects model was employed.

**Figure 4:**
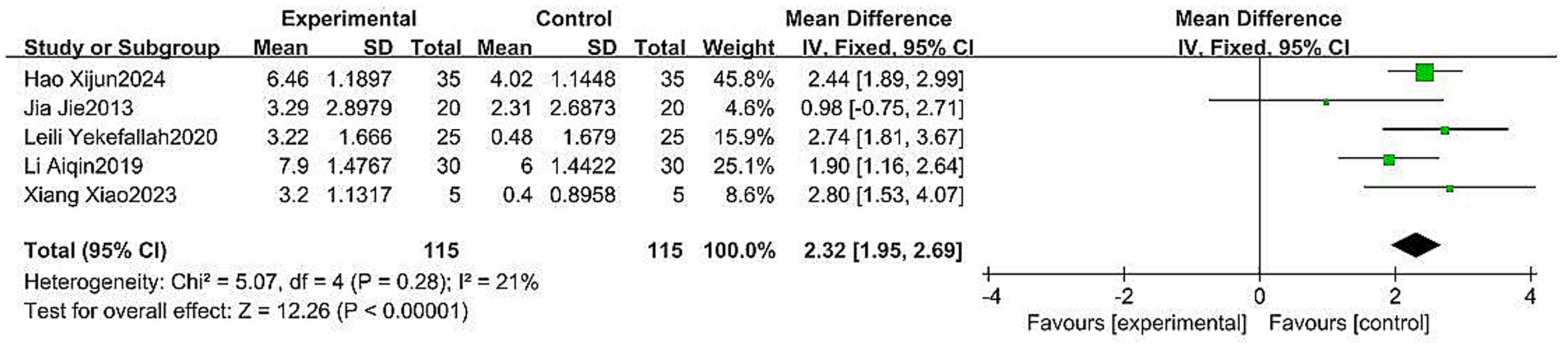
Forest plot of the effect of music intervention on continuous GCS scores in patients with disorders of consciousness (DOC).

The pooled results demonstrated that music intervention significantly increased GCS scores (MD = 2.32, 95% CI: 1.95–2.69, *Z* = 12.26, *p* < 0.00001) (Figure 4).

**Figure 5:**
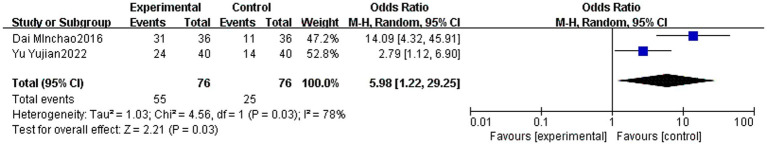
Forest plot of the effect of music intervention on the proportion of patients achieving a GCS score of > 8 in disorders of consciousness (DOC).

The binary analysis with a GCS score of > 8 as the outcome included two studies, with 76 patients in the experimental group and 76 in the control group. Heterogeneity testing indicated high inter-study heterogeneity (*χ*^2^ = 4.56, df = 1, *p* = 0.03; *I*^2^ = 78%); therefore, a random-effects model was used. The pooled results showed that music intervention significantly increased the proportion of patients achieving a GCS score > 8 (OR = 5.98, 95% CI: 1.22–29.25, Z = 2.21, *p* = 0.03)(Figure 5). Given the small number of included studies for this outcome, the high inter-study heterogeneity, and the wide 95% CI, the results should still be interpreted with caution.

**Figure 6:**
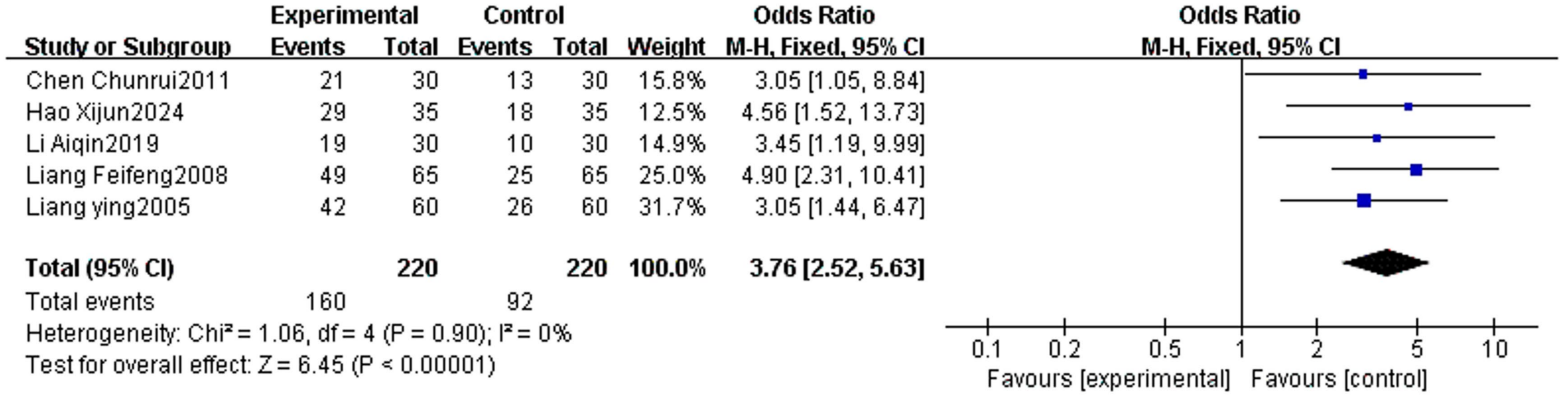
Forest plot of the effect of music intervention on the awakening rate in patients with disorders of consciousness (DOC).

The analysis of the awakening rate included five studies, with 220 patients in the experimental group and 220 in the control group. Heterogeneity testing showed no significant heterogeneity (*χ*^2^ = 1.06, df = 4, *p* = 0.90; *I*^2^ = 0%); therefore, a fixed-effects model was employed. The pooled results indicated that music intervention significantly improved the awakening rate (OR = 3.76, 95% CI: 2.52–5.63, Z = 6.45, *p* < 0.00001) (Figure 6).

#### 3.3.2 Effects of music intervention on the CRS-R

**Figure 7:**
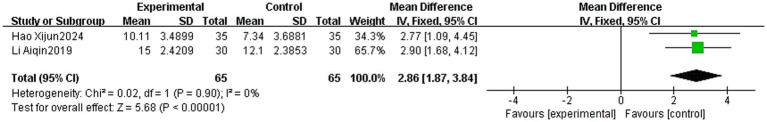
Forest plot of the effect of music intervention on Coma Recovery Scale-Revised (CRS-R) scores.

A total of two studies reported post-intervention CRS-R scores, with 65 patients in the experimental group and 65 in the control group. Heterogeneity testing showed no significant inter-study heterogeneity (*χ*^2^ = 0.02, df = 1, *p* = 0.90; *I*^2^ = 0%); therefore, a fixed-effects model was used for the pooled analysis. The pooled results demonstrated that music intervention significantly improved CRS-R scores (MD = 2.86, 95% CI: 1.87–3.84, Z = 5.68, *p* < 0.00001), suggesting that music intervention may facilitate consciousness recovery (Figure 7). Given that only two studies were included for this outcome, the leave-one-out sensitivity analysis was not displayed separately. Due to the limited sample size, these results should be interpreted with caution.

#### 3.3.3 Effects of music intervention on the degree of neurological deficit (NIHSS)

**Figure 8:**
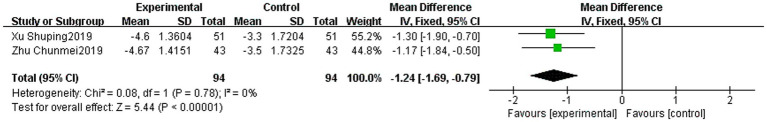
Forest plot of the effect of music intervention on the degree of neurological deficit (NIHSS).

A total of two studies reported post-intervention NIHSS scores, with 94 patients in the experimental group and 94 in the control group. Heterogeneity testing showed no significant inter-study heterogeneity (*χ*^2^ = 0.08, df = 1, *p* = 0.78; *I*^2^ = 0%); therefore, a fixed effects model was used for the pooled analysis. The pooled results showed that NIHSS scores in the music intervention group were significantly lower than those in the control group (MD = −1.24, 95% CI: −1.69 to −0.79, Z = 5.44, *p* < 0.00001) (Figure 8). Although the NIHSS is not designed to directly evaluate a patient’s clarity of consciousness or level of arousal, it is primarily used to reflect the degree of neurological deficit, with lower scores typically indicating less severe neurological impairment. Therefore, the NIHSS results can serve as a supplementary outcome alongside awakening improvement, helping to determine whether music intervention is accompanied by an improvement in the patient’s neurological status. In the present study, the reduction in NIHSS scores in the music intervention group suggests a positive effect on alleviating certain neurological deficits. Given that this outcome was based on only a few studies, the relevant sensitivity analysis was not displayed separately, and the results should be interpreted with caution.

#### 3.3.4 GRADE quality of evidence analysis of music intervention for disorders of consciousness

**Figure 9:**
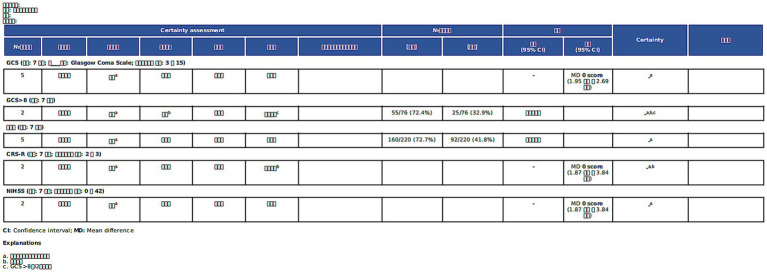
GRADE assessment of the certainty of evidence for music intervention in patients with disorders of consciousness (DOC).

In this study, the GRADE approach was used to assess the certainty of evidence for the five outcomes included in the meta-analysis; the results are shown in Figure 9. Overall, the certainty of evidence for the GCS, awakening rate, CRS-R, and NIHSS was moderate, while the certainty of evidence for a GCS score of > 8 was low.

The GCS outcome included a total of five studies. The meta-analysis results showed that the music intervention group had significantly higher GCS scores than the control group (MD = 2.32, 95% CI: 1.95–2.69, *I*^2^ = 21%). According to the GRADE assessment, the certainty of evidence for this outcome was moderate.

The GCS score of > 8 outcome included a total of two studies. The meta-analysis results showed that the proportion of patients with a GCS score of > 8 in the music intervention group was higher than that in the control group (OR = 5.98, 95% CI: 1.22–29.25, *I*^2^ = 78%). According to the GRADE assessment, the certainty of evidence for this outcome was low.

The awakening rate outcome included a total of five studies. The meta-analysis results showed that the awakening rate in the music intervention group was higher than that in the control group (OR = 3.76, 95% CI: 2.52–5.63, *I*^2^ = 0%). According to the GRADE assessment, the certainty of evidence for this outcome was moderate.

The CRS-R outcome included a total of two studies. The meta-analysis results showed that CRS-R scores in the music intervention group were higher than those in the control group (MD = 2.86, 95% CI: 1.87–3.84, *I*^2^ = 0%). According to the GRADE assessment, the certainty of evidence for this outcome was moderate.

The NIHSS outcome included a total of two studies. The meta-analysis results showed that NIHSS scores in the music intervention group were lower than those in the control group (MD = −1.24, 95% CI: −1.69 to −0.79, *I*^2^ = 0%). According to the GRADE assessment, the certainty of evidence for this outcome was moderate.

In summary, among the five outcomes evaluated, the certainty of evidence for the GCS, awakening rate, CRS-R, and NIHSS was moderate, while the certainty of evidence for a GCS score > 8 was low. Overall, current evidence suggests that music intervention has a positive effect on several clinical outcomes in patients with disorders of consciousness (DOC); however, outcomes with low certainty of evidence should be interpreted with caution.

## 4 Discussion

This systematic review included 12 randomized controlled trials (RCTs) involving 926 patients with disorders of consciousness (DOC). As the outcome indicators reported by the included studies differed, each meta-analysis was conducted based on studies that reported the corresponding outcome and provided extractable data. The results indicated that music intervention had a positive effect on improving arousal levels and awakening outcomes, as evidenced by the findings for GCS scores, awakening rates, and CRS-R scores. Furthermore, although the NIHSS does not directly reflect the level of consciousness, it serves as an assessment tool for the severity of neurological deficit. The findings, therefore, suggest that music intervention may also have a positive effect on alleviating certain neurological impairments. It should be noted that, although the outcome of a GCS score of > 8 also reached statistical significance, these results should be interpreted with caution due to the small number of included studies and the high inter-study heterogeneity. To further explain the positive findings observed in GCS scores, awakening rates, and CRS-R outcomes in this study, the following section discusses the potential mechanisms of music intervention in light of previous mechanistic studies.

The pooled results indicated that the music intervention group had an advantage in awakening outcomes, suggesting that its effects may not be limited to the short-term behavioral responses elicited during stimulation itself, but they are more likely associated with sustained improvements in consciousness over time. It should be noted that this judgment is primarily based on clinical outcomes; the value of previous mechanism-related studies lies more in providing consistent supplementary evidence rather than directly establishing a causal relationship. Existing studies have found that musical stimulation can be accompanied by behavioral and neurophysiological responses (O’Kelly et al., 2013), indicating that some patients with disorders of consciousness (DOC) can exhibit measurable overt or physiological changes after receiving musical input. Other research has observed autonomic nervous system responses (Li et al., 2021), suggesting that musical stimulation may influence physiological regulation states related to arousal, thereby providing indirect support for clinical improvement. At the electrophysiological level, studies have reported detectable electroencephalography (EEG) responses in DOC patients during name-calling and musical stimulation (Bai et al., 2013), suggesting that some patients retain basic processing capabilities for external auditory input. Furthermore, research has found that individualized musical stimulation can induce theta–gamma coupling characteristics (Xiao et al., 2024), providing indirect evidence that musical stimulation may involve higher-level information integration processes. In addition, consciousness assessment studies based on musical stimulation paradigms (Pan et al., 2024) and auditory stimulation-related brain activation studies (Qu et al., 2024) also indicate that music-related stimuli help identify patients’ residual processing capabilities. At the level of brain function, studies on resting-state functional connectivity (Boltzmann et al., 2021), brain functional characteristics and awakening prediction (Hu, 2018), and the relationship between music therapy and activity in different brain regions (Steinhoff et al., 2015) provide biological plausibility for the potential influence of music intervention on consciousness recovery from the perspectives of network and regional brain activity.

However, a majority of these studies are based on small samples and involve inconsistent indicators, paradigms, and analysis methods; therefore, they are currently more suitable as supplementary explanations for clinical results rather than constituting primary conclusions on their own. The aforementioned mechanistic studies may provide a certain explanatory basis for the clinical outcomes of this study; however, because this evidence is largely derived from small-sample or mechanistic studies, it cannot directly prove the causal pathway through which music intervention improves consciousness recovery. The primary conclusions of this study should still be based on the pooled clinical outcome results of the included randomized controlled trials.

Although the pooled directions of a majority of outcomes were generally consistent, the levels of heterogeneity varied across outcomes. Specifically, the GCS score of > 8 outcome exhibited relatively high inter-study heterogeneity, suggesting that its pooled results may be more susceptible to differences in the original study designs and intervention content; therefore, further discussion is necessary. For the GCS score of > 8 outcome, two studies were included, one of which was conducted by Dai et al. (2016) using a three-arm randomized controlled design. According to the encoding principles of this study, this research can be interpreted as consisting of two music medicine experimental arms and one routine treatment control arm: Experimental arm 1 involved patient-preferred music playback, experimental arm 2 involved specific music playback, and the control arm involved routine treatment. The results presented a clear gradient relationship, with the patient-preferred music group showing the greatest effect, followed by the specific music group, while the routine treatment group showed the weakest effect. This finding suggests that the efficacy of music intervention depends not only on the application of musical stimulation but, more importantly, on the degree of matching between musical materials and the patient’s past experiences. The patient-preferred music group is more closely associated with the patient’s life experiences, familiarity, and emotional memory, making it more likely to activate preserved autobiographical memory and affective processing pathways (Grimm and Kreutz, 2018). In contrast, although the latter provides rhythmic and melodic stimulation, its effect may be limited to the level of general arousal and auditory input if it lacks clear individual relevance.

Furthermore, the intervention format may significantly influence therapeutic efficacy. Among the included studies, only one by Xiao et al. (2023) met the operational definition of music therapy, as it was implemented on-site by a music therapist within a therapeutic relationship and involving real-time adjustments based on patient responses. Most of the remaining studies were playback-based interventions without the involvement of a music therapist. Since only this single study could be categorized as music therapy, it was not possible to conduct subgroup analyses to compare differences in effect sizes between music therapy and music medicine.

However, the forest plot results show that the effect size reported in this study was superior to a majority of studies involving music playback alone, suggesting that therapist involvement and live interaction may have an additive effect on efficacy. Nevertheless, current evidence is insufficient to draw definitive conclusions that “music therapy outcomes are superior to other music presentation formats” or “live music results are superior to recorded playback.” On the one hand, the relevant evidence is derived from only a single study with a limited sample size, which does not provide a sufficient basis for systematic comparison. On the other hand, therapist intervention itself introduces numerous complex factors, including the therapist’s professional background and experience level, the specific implementation methods, the atmosphere and rhythm of live interactions, real-time response strategies based on the patient’s state, and the criteria for judging efficacy.

The primary significance of this study lies in the systematic synthesis of the effects of music intervention in the field of disorders of consciousness (DOC) based on evidence from randomized controlled trials (RCTs), with separate analyses conducted for key clinical outcomes such as the GCS, GCS score of > 8, awakening rate, CRS-R, and NIHSS. Consequently, this study not only further supports the potential effectiveness of music intervention but also further clarifies the specific outcome measures showing positive effects based on a higher level of evidence; simultaneously, it identifies research findings that continue to necessitate cautious interpretation in the context of heterogeneity.

This study has several limitations. First, the number of included studies was limited, and some outcomes were based on only a small number of studies, which may have affected the robustness of the pooled results. The clinical states of the included patients also varied, including coma, minimally conscious state, and studies that did not clearly distinguish specific DOC subtypes. Since coma, VS/UWS, and MCS differ in prognosis, disease stage, and pathophysiological mechanisms, pooled analysis may introduce clinical heterogeneity. Due to the limited number of studies and insufficient reporting in the original studies, subgroup analyses by DOC subtype could not be performed.

Second, the use of standardized assessment tools remained insufficient. Although the CRS-R is important for assessing disorders of consciousness and distinguishing UWS from MCS, only two studies reported CRS-R scores. A majority of studies continued to use the GCS, awakening rates, or a GCS scores of > 8 to evaluate intervention effects. The NIHSS primarily reflects neurological deficits and is not specifically designed to assess consciousness recovery. Therefore, the pooled results for the CRS-R and NIHSS should be considered exploratory and should be interpreted with caution.

Third, the included studies differed in music type, intervention frequency, intervention duration, assessment time points, patient etiology, disease stage, and baseline consciousness level, which may have influenced the pooled results. Since the original studies insufficiently reported sample characteristics, music types, stimulus sources, and treatment formats, further subgroup analyses could not be conducted. Fourth, a majority of studies used receptive interventions based on simple music playback, and few studies directly compared preferred music with ordinary music; therefore, the effects of music type and therapist involvement remain difficult to determine.

Future studies should conduct larger-sample, multicenter, and rigorously designed randomized controlled trials, with clear sample size calculations, random allocation, allocation concealment, and blinded outcome assessment. Patients’ DOC subtype, etiology, disease stage, and baseline consciousness level should be clearly reported, and stratified analyses should be conducted when the sample size permits. Standardized assessment tools such as the CRS-R should be prioritized, and main outcomes, assessment time points, and follow-up duration should be unified to the greatest extent possible. Intervention protocols should further clarify music type, basis for music selection, stimulus source, intervention frequency, session duration, and total treatment course to improve comparability across studies and the stability of result interpretation.

## 5 Conclusion

Based on evidence from randomized controlled trials (RCTs), this systematic review and meta-analysis systematically evaluated the efficacy of music intervention in patients with disorders of consciousness (DOC). The results showed that music intervention led to statistically significant improvements in outcome measures, including the Glasgow Coma Scale (GCS), awakening rate, and Coma Recovery Scale-Revised (CRS-R), suggesting a positive effect on patients’ arousal levels and awakening outcomes. Simultaneously, although the NIHSS does not directly reflect the level of consciousness, the results suggest that music intervention may have a positive effect on alleviating certain neurological deficits in patients.

Although the pooled result for a GCS score of > 8 was also significant, this conclusion should be interpreted with caution due to the high inter-study heterogeneity. Overall, current evidence supports the general effectiveness of music intervention while suggesting possible variations in efficacy across different music types and implementation methods. As a non-invasive and safe adjunctive modality, music intervention demonstrates considerable application value in the rehabilitation of patients with disorders of consciousness (DOC). However, given the limited number of included studies, the heterogeneity of intervention protocols, and the inconsistency in outcome measures, its true efficacy remains to be further validated. Future research should rely on larger-sample, rigorously designed randomized controlled trials (RCTs) to deepen the understanding of both the clinical effects and the underlying mechanisms of music intervention.

## Data Availability

The original contributions presented in the study are included in the article/Supplementary material; further inquiries can be directed to the corresponding author.
