# Supplementary material for: Effects of music interventions on disorders of consciousness: a systematic review and meta-analysis
Source: Front Neurosci. 2026 Jul 24;20:1843996. doi: 10.3389/fnins.2026.1843996 (PMC13447351; doi:10.3389/fnins.2026.1843996)
Supplement: Supplementary file 1 [file Table_1.DOCX]

| **Table S1** The search strategy | | |
| --- | --- | --- |
| **Datebases** | **Searching strategy** | **Literature number** |
| Pubmed | ("Music"[MeSH Terms] OR "Music Therapy"[MeSH Terms] OR "Music"[Title/Abstract] OR "Music Therapy"[Title/Abstract] OR "Therapy, Music"[Title/Abstract] OR "Songs"[Title/Abstract] OR "Song"[Title/Abstract] OR "Vocal Melody"[Title/Abstract] OR "Melodies, Vocal"[Title/Abstract] OR "Melody, Vocal"[Title/Abstract] OR "Vocal Melodies"[Title/Abstract] OR "Classical Music"[Title/Abstract] OR "Music, Classical"[Title/Abstract] OR "Jazz Music"[Title/Abstract] OR "Music, Jazz"[Title/Abstract] OR "Rap Music"[Title/Abstract] OR "Music, Rap"[Title/Abstract] OR "Hip Hop Music"[Title/Abstract] OR "Hop Music, Hip"[Title/Abstract] OR "Music, Hip Hop"[Title/Abstract] OR "Rock and Roll Music"[Title/Abstract]) AND ("Consciousness Disorders"[MeSH Terms] OR "Consciousness Disorder"[Title/Abstract] OR "Consciousness Disorders"[Title/Abstract] OR "Disorders of Consciousness"[Title/Abstract] OR "Disorder of Consciousness"[Title/Abstract] OR "Consciousness, Level Depressed"[Title/Abstract] OR "Depressed Level of Consciousness"[Title/Abstract] OR "Semiconsciousness"[Title/Abstract] OR "Consciousness, Level Altered"[Title/Abstract] OR "Altered Level of Consciousness"[Title/Abstract] OR "Coma"[MeSH Terms] OR "Coma"[Title/Abstract] OR "Comas"[Title/Abstract] OR "Comatose"[Title/Abstract] OR "Pseudocoma"[Title/Abstract] OR "Pseudocomas"[Title/Abstract] OR "Persistent Vegetative State"[MeSH Terms] OR "PVS"[Title/Abstract] OR "PVSs"[Title/Abstract] OR "Persistent Vegetative State"[Title/Abstract] OR "Persistent Vegetative States"[Title/Abstract] OR "Vegetative State, Persistent"[Title/Abstract] OR "Vegetative States, Persistent"[Title/Abstract] OR "Permanent Vegetative State"[Title/Abstract] OR "Permanent Vegetative States"[Title/Abstract] OR "Vegetative State, Permanent"[Title/Abstract] OR "Vegetative States, Permanent"[Title/Abstract] OR "Vegetative State"[Title/Abstract] OR "Vegetative States"[Title/Abstract] OR "Transient Vegetative State"[Title/Abstract] OR "Transient Vegetative States"[Title/Abstract] OR "Minimally Conscious State"[Title/Abstract] OR "Minimally Conscious States"[Title/Abstract] OR "MCS"[Title/Abstract] OR "Unresponsive Wakefulness Syndrome"[Title/Abstract] OR "UWS"[Title/Abstract]) | **175** |
| Web of Science | #1：TS=("Consciousness Disorders" OR "Consciousness Disorder" OR "Disorders of Consciousness" OR "Disorder of Consciousness" OR "Consciousness, Level Depressed" OR "Depressed Level of Consciousness" OR "Semiconsciousness" OR "Consciousness, Level Altered" OR "Altered Level of Consciousness" OR "Coma" OR "Comas" OR "Comatose" OR "Pseudocoma" OR "Pseudocomas" OR "Persistent Vegetative State" OR "PVS" OR "PVSs" OR "Persistent Vegetative States" OR "Vegetative State, Persistent" OR "Vegetative States, Persistent" OR "Permanent Vegetative State" OR "Permanent Vegetative States" OR "Vegetative State, Permanent" OR "Vegetative States, Permanent" OR "Vegetative State" OR "Vegetative States" OR "Transient Vegetative State" OR "Transient Vegetative States" OR "Minimally Conscious State" OR "Minimally Conscious States" OR "MCS" OR "Unresponsive Wakefulness Syndrome" OR "UWS")  #2：TS=("Music" OR "Music Therapy" OR "Therapy, Music" OR "Rap Music" OR "Music, Rap" OR "Hip Hop Music" OR "Hop Music, Hip" OR "Music, Hip Hop" OR "Jazz Music" OR "Music, Jazz" OR "Classical Music" OR "Music, Classical" OR "Songs" OR "Song" OR "Vocal Melody" OR "Melodies, Vocal" OR "Melody, Vocal" OR "Vocal Melodies" OR "Rock and Roll Music")  #3： #1 AND #2  Timespan:1970-01-01 to 2025-12-31 (Publication Date) | **160** |
| Embase | **#61 #60 AND #23 491**  **#60 #59 OR #58 OR #57 OR #56 OR #55 OR #54 OR #53 OR #52 OR #51 OR #50 OR #49 OR #48 OR #47 OR #46 OR #45 OR #44 OR #43 OR #42 OR #41 OR #40 OR #39 OR #38 OR #37 OR #36 OR #35 OR #34 OR #33 OR #32 OR #31 OR #30 OR #28 OR #29 OR #27 OR #26 OR #25 OR #24 261,086**  **#59 'uws':ab,ti 1,127**  **#58 'unresponsive wakefulness syndrome':ab,ti 849**  **#57 'mcs':ab,ti 31,904**  **#56 'minimally conscious states':ab,ti 219**  **#55 'minimally conscious state':ab,ti 2,036**  **#54 'transient vegetative states':ab,ti**  **#53 'transient vegetative state':ab,ti 1**  **#52 'vegetative states':ab,ti 195**  **#51 'vegetative state':ab,ti 4,750**  **#50 'vegetative states, permanent':ab,ti**  **#49 'vegetative state, permanent':ab,ti 1**  **#48 'permanent vegetative states':ab,ti 6**  **#47 'permanent vegetative state':ab,ti 181**  **#46 'vegetative states, persistent':ab,ti**  **#45 'vegetative state, persistent':ab,ti 2**  **#44 'persistent vegetative states':ab,ti 62**  **#43 'persistent vegetative state':ab,ti 1,329**  **#42 'pvss':ab,ti 217**  **#41 'pvs':ab,ti 12,172**  **#40 'pseudocomas':ab,ti**  **#39 'pseudocoma':ab,ti 14**  **#38 'comatose':ab,ti 9,653**  **#37 'comas':ab,ti 762**  **#36 'coma':ab,ti 62,582**  **#35 'altered level of consciousness':ab,ti 1,073**  **#34 'consciousness, level altered':ab,ti**  **#33 'semiconsciousness':ab,ti 30**  **#32 'depressed level of consciousness':ab,ti 315**  **#31 'consciousness, level depressed':ab,ti**  **#30 'disorder of consciousness':ab,ti 771**  **#29 'disorders of consciousness':ab,ti 3,967**  **#28 'consciousness disorders':ab,ti 687**  **#27 'consciousness disorder':ab,ti 438**  **#26 'persistent vegetative state'/exp 6,338**  **#25 'coma'/exp 74,335**  **#24 'consciousness disorders'/exp 176,179**  **#23 #1 OR #2 OR #3 OR #4 OR #5 OR #6 OR #7 OR #8 OR #9 OR #10 OR #11 OR #12 OR #13 OR #14 OR #15 OR #16 OR #17 OR #18 OR #19 OR #20 OR #21 OR #22 59,362**  **#22 'rock and roll music':ti,ab 19**  **#21 'rock and roll music':ti,ab 19**  **#20 'music, hip hop':ti,ab 1**  **#19 'hop music, hip':ti,ab**  **#18 'hip hop music':ti,ab 36**  **#17 'music, rap':ti,ab 8**  **#16 'rap music':ti,ab 93**  **#15 'music, jazz':ti,ab 8**  **#14 'jazz music':ti,ab 41**  **#13 'music, classical':ti,ab 54**  **#12 'classical music':ti,ab 900**  **#11 'vocal melodies':ti,ab 12**  **#10 'melody, vocal':ti,ab 3**  **#9 'melodies, vocal':ti,ab**  **#8 'vocal melody':ti,ab 5**  **#7 'song':ti,ab 11,894**  **#6 'songs':ti,ab 3,980**  **#5 'therapy, music':ti,ab 303**  **#4 'music therapy':ti,ab 6,915**  **#3 'music':ti,ab 35,956**  **#2 'music'/exp 30,494**  **#1 'music therapy'/exp 12,599**  **Date: 1 Jul 2026** | **491** |
| Cochrane Library | **#1 MeSH descriptor: [Music] explode all trees**  **#2 MeSH descriptor: [Music Therapy] explode all trees**  **#3 "Music":ti,ab,kw OR "Music Therapy":ti,ab,kw OR "Therapy, Music":ti,ab,kw OR "Music Intervention":ti,ab,kw OR "Music Interventions":ti,ab,kw OR "Music Listening":ti,ab,kw OR "Listening to Music":ti,ab,kw OR "Songs":ti,ab,kw OR "Song":ti,ab,kw OR "Singing":ti,ab,kw OR "Vocal Music":ti,ab,kw OR "Vocal Melody":ti,ab,kw OR "Classical Music":ti,ab,kw OR "Jazz Music":ti,ab,kw OR "Rap Music":ti,ab,kw OR "Hip Hop Music":ti,ab,kw OR "Rock Music":ti,ab,kw OR "Rock and Roll Music":ti,ab,kw OR "Rhythm":ti,ab,kw OR "Rhythmic Auditory Stimulation":ti,ab,kw**  **#4 #1 OR #2 OR #3**  **#5 MeSH descriptor: [Consciousness Disorders] explode all trees**  **#6 MeSH descriptor: [Coma] explode all trees**  **#7 MeSH descriptor: [Persistent Vegetative State] explode all trees**  **#8 "Consciousness Disorders":ti,ab,kw OR "Consciousness Disorder":ti,ab,kw OR "Disorders of Consciousness":ti,ab,kw OR "Disorder of Consciousness":ti,ab,kw OR "Altered Level of Consciousness":ti,ab,kw OR "Depressed Level of Consciousness":ti,ab,kw OR "Disturbance of Consciousness":ti,ab,kw OR "Impaired Consciousness":ti,ab,kw OR "Reduced Consciousness":ti,ab,kw OR "Semiconsciousness":ti,ab,kw OR "Coma":ti,ab,kw OR "Comas":ti,ab,kw OR "Comatose":ti,ab,kw OR "Pseudocoma":ti,ab,kw OR "Persistent Vegetative State":ti,ab,kw OR "Persistent Vegetative States":ti,ab,kw OR "Vegetative State":ti,ab,kw OR "Vegetative States":ti,ab,kw OR "Permanent Vegetative State":ti,ab,kw OR "Transient Vegetative State":ti,ab,kw OR "Minimally Conscious State":ti,ab,kw OR "Minimally Conscious States":ti,ab,kw OR "Unresponsive Wakefulness Syndrome":ti,ab,kw OR "UWS":ti,ab,kw OR "PVS":ti,ab,kw OR "MCS":ti,ab,kw**  **#9 #5 OR #6 OR #7 OR #8**  **#10 #4 AND #9** | **290** |
| CNKI | SU=('音乐'*'意识障碍') OR SU=('音乐'*'昏迷') OR SU=('音乐'*'持续性植物状态') OR SU=('音乐'*'植物状态') OR SU=('音乐'*'微意识状态') OR SU=('音乐治疗'*'意识障碍') OR SU=('音乐治疗'*'昏迷') OR SU=('音乐治疗'*'持续性植物状态') OR SU=('音乐治疗'*'植物状态') OR SU=('音乐治疗'*'微意识状态') OR (TI='音乐' OR TI='音乐治疗' OR TI='歌曲' OR TI='声乐旋律' OR TI='古典音乐' OR TI='爵士乐' OR TI='说唱音乐' OR TI='嘻哈音乐' OR TI='摇滚乐') AND (TI='意识障碍' OR TI='昏迷' OR TI='持续性植物状态' OR TI='植物状态' OR TI='微意识状态' OR TI='无反应觉醒综合征' OR TI='PVS' OR TI='MCS' OR TI='UWS') OR (KY='音乐' OR KY='音乐治疗' OR KY='歌曲' OR KY='声乐旋律' OR KY='古典音乐' OR KY='爵士乐' OR KY='说唱音乐' OR KY='嘻哈音乐' OR KY='摇滚乐') AND (KY='意识障碍' OR KY='昏迷' OR KY='持续性植物状态' OR KY='植物状态' OR KY='微意识状态' OR KY='无反应觉醒综合征' OR KY='PVS' OR KY='MCS' OR KY='UWS') OR (AB='音乐' OR AB='音乐治疗' OR AB='歌曲' OR AB='声乐旋律' OR AB='古典音乐' OR AB='爵士乐' OR AB='说唱音乐' OR AB='嘻哈音乐' OR AB='摇滚乐') AND (AB='意识障碍' OR AB='昏迷' OR AB='持续性植物状态' OR AB='植物状态' OR AB='微意识状态' OR AB='无反应觉醒综合征' OR AB='PVS' OR AB='MCS' OR AB='UWS') | **221** |
| Proquest | #1: SUMMARY("Consciousness Disorders" OR "Consciousness Disorder" OR "Disorders of Consciousness" OR "Disorder of Consciousness" OR "Consciousness, Level Depressed" OR "Depressed Level of Consciousness" OR "Semiconsciousness" OR "Consciousness, Level Altered" OR "Altered Level of Consciousness" OR "Coma" OR "Comas" OR "Comatose" OR "Pseudocoma" OR "Pseudocomas" OR "Persistent Vegetative State" OR "PVS" OR "PVSs" OR "Persistent Vegetative States" OR "Vegetative State, Persistent" OR "Vegetative States, Persistent" OR "Permanent Vegetative State" OR "Permanent Vegetative States" OR "Vegetative State, Permanent" OR "Vegetative States, Permanent" OR "Vegetative State" OR "Vegetative States" OR "Transient Vegetative State" OR "Transient Vegetative States" OR "Minimally Conscious State" OR "Minimally Conscious States" OR "MCS" OR "Unresponsive Wakefulness Syndrome" OR "UWS")  #2: SUMMARY("Music" OR "Music Therapy" OR "Therapy, Music" OR "Rap Music" OR "Music, Rap" OR "Hip Hop Music" OR "Hop Music, Hip" OR "Music, Hip Hop" OR "Jazz Music" OR "Music, Jazz" OR "Classical Music" OR "Music, Classical" OR "Songs" OR "Song" OR "Vocal Melody" OR "Melodies, Vocal" OR "Melody, Vocal" OR "Vocal Melodies" OR "Rock and Roll Music")  #3: #1 AND #2  Timespan: 1970-01-01 to 2025-12-3 | **41** |
